# Infant Social Development across the Transition from Crawling to Walking

**DOI:** 10.3389/fpsyg.2016.00960

**Published:** 2016-06-27

**Authors:** Eric A. Walle

**Affiliations:** Psychological Sciences, University of California, MercedMerced, CA, USA

**Keywords:** social development, language, motor development, walking, joint attention

## Abstract

The onset of walking is a developmental transition that sets in motion a cascade of change across a range of domains, including social interactions and language learning. However, research on the unfolding of such change in the infant across this transition is limited. This investigation utilized a longitudinal design to examine the effect of walking acquisition on infant social development and parent perceptions of the infant to explore how changes in these factors relate with infant language development. Parents reported on infant social behaviors and their perception of the infant, as well as motor and language development, in 2-week intervals from 10.5 to 13 months of age. Mixed linear models revealed infant initiation of joint engagement (e.g., pointing, bringing objects to the parent) and following of the parent's joint engagement cues (e.g., point following, gaze following) increased as a function of infant walking experience, particularly between 2- and 4-weeks after the onset of walking, independent of age. Additionally, the parent's perception of the infant as an individual increased between 2- and 4-weeks after the infant began to walk. Finally, the unique relations of infant walking experience, following of social cues, and the parents' perception of the infant as an individual with infant language development were examined. Infant following of joint engagement behaviors and parent perception of the infant as an individual were related to receptive, but not productive, vocabulary size. Additionally, infant walking experience remained a significant predictor of infant receptive and productive language. These findings provide insight on important factors that change as the infant begins to walk. Future research utilizing more direct assessment of these factors is described, as well as general patterning of developmental change across the transition from crawling to walking.

## Motor development as a facilitator of change

There is a rich, albeit often overlooked, theoretical literature viewing development as a reciprocal and non-linear process of change. Gottlieb's (1983) emphasis on epigenesis highlights the bidirectional and transactional nature of development. Both classic and contemporary theorizing has similarly argued for a bidirectional and dynamic framework of development (e.g., Gibson, 1958; Thelen, 1995; Bernstein, 1996; Thelen and Smith, 1998) in which particular experiences serve as catalysts for developmental cascades (e.g., Spencer et al., 2009; Masten and Cicchetti, 2010). Infant motor development is one particular skill that generates a host of new experiences and is associated with changes in psychological functioning across a broad range of domains (for reviews, see Campos et al., 2000; Iverson, 2010). In particular, a growing body of research indicates that the transition from crawling to walking corresponds with an increase in infant receptive and productive language (Oudgenoeg-Paz et al., 2012; Walle and Campos, 2014; He et al., 2015).

Although this research has demonstrated a clear association between infant motor and linguistic development, it has fallen short in elucidating the mechanisms that account for this developmental change. More concretely, it is unlikely that the acquisition of upright locomotion *per se* causes infants to develop language, just as it is unlikely that infant language causes the onset of walking. Rather, the onset of walking likely corresponds with changes in a broad range of domains, including, but not limited to, language. Research exploring the developmental trajectories of different abilities across the transition from crawling to walking may elucidate the underlying mechanism(s) that account for the relation between walking and language. However, the only longitudinal study to investigate the association between infant walking and language (Walle and Campos, 2014) did not explore potential processes that may account for this link.

The present longitudinal investigation utilized parent report of changes in the infant's following and solicitation of joint engagement, the parent's perceptions of their infant, the infant's language development, and locomotor development. Assessing these abilities in 2-week intervals from 10.5 to 13 months of age permitted the close examination of how each changed across the transition from crawling to walking.

### Parent and infant joint engagement

The acquisition of an upright posture increases the infant's visual field (Kretch et al., 2014) and permits greater flexibility with which to view the environment (Frank et al., 2013). These physical changes may promote infant following of adult attentional cues, and thereby facilitate language learning. Engaging in joint attention behavior is essential for the development of language (Tomasello, 1988, 1995). Such episodes of joint engagement occur when one individual directs the attention of another to a shared referent, such as an object or event. Multiple studies have found that infant following of adult attentional cues is related to language development (Tomasello and Todd, 1983; Smith et al., 1988; Mundy et al., 1995; Morales et al., 1998; Brooks and Meltzoff, 2005). Likewise, infant initiation of joint engagement, such as pointing, is also associated with subsequent language development (Brooks and Meltzoff, 2008; LeBarton et al., 2015). Perhaps not surprisingly, infant joint attention, particularly following adult gaze, also develops markedly following the infant's first birthday (Morissette et al., 1995; Morales et al., 2000), when infants typically begin to walk. However, existing longitudinal research of infant following of joint attention cues has not examined how this ability is impacted by the onset of walking.

Furthermore, infant walking also has a significant impact on how the infant engages with the caregiver. Walking infants are reported by parents as more willful (Biringen et al., 1995) and have been observed to be more likely to access objects located further away than crawling infants (Clearfield, 2011; Karasik et al., 2011). Additionally, engaging in mobile bids for the parent's attention, such as carrying an object to the parent, elicits more interactive, and verbally rich responses by the parent and such bids are more frequent by walking than crawling infants (Karasik et al., 2014). Walking infants have also been found to direct the parent's attention to objects using vocalizations and gestures more than crawling infants (Clearfield et al., 2008; Clearfield, 2011; Karasik et al., 2011). These findings indicate that not only may the walking infant be more attuned to follow adult attentional cues, but they also help to generate social contexts in which they themselves elicit parent attention. However, prior longitudinal research has not examined how such changes in infant elicitation of parent attention across this developmental transition is related with infant language development.

The above research indicates that walking infants initiate and engage in richer joint engagement interactions in social contexts than crawling infants. Changes in infants' ability to engage with the environment combined with corresponding changes in parents' responding may help to facilitate infant language acquisition. However, previous research has not examined the developmental trajectories of these skills across the transition from crawling to walking.

### Parent perception of the infant

The acquisition of walking may also change how parents perceive their infants. How parents perceive their infant has a profound impact on their inferences about infants' behavior (Rubin et al., 1974), interactions with their infants (Will et al., 1976), and expectations of likely actions (Mondschein et al., 2000). Upright locomotion is a uniquely human characteristic. By contrast, a crawling infant is more akin in physical appearance to a quadrupedal animal. Adult attribution of greater intentionality and responsibility to walking infants' than crawling infants may impact infant vocabulary size in two ways.

First, parents may interact differently with their infant if they perceive their infant as more intentional and human-like. Such differences in interactive style may promote specific behaviors and social responses when engaging with the infant, as suggested by parents' differential reinforcement of more speech-like babbling (Warlaumont et al., 2014). Thus, parents who believe that their infant is a more capable interactive partner may provide qualitatively different communication. Second, prior research linking infant walking and language has relied on parent reporting of infant receptive and productive vocabularies. Although the MacAruthur-Bates Communicative Development Inventory (MCDI) is a commonly used and validated measure (see Fenson et al., 1994, 2000; Ring and Fenson, 2000), it is possible that the parents attributing greater linguistic skill to walking infants over crawling infants may inadvertently inflate their vocabulary sizes. For example, a crawling and a walking infant might both utter the same vocalization (e.g., du-ga-ga). The parent's perception of the walking as more human-like may result in the parent attributing greater intentionality to this behavior and conclude that the child was verbalizing (e.g., doggy), whereas the same vocalization by the crawling infant may be dismissed as babbling. Thus, it is possible that previously reported differences in crawling and walking infants' vocabulary sizes is attributable to parents' differential appraisals of their infant's proficiency, not an objective change in language development. Accounting for parents' perception of the infant as an intentional individual when analyzing parent reporting of infant behavior is essential to help rule out this alternative explanation.

## The present study

No study to the author's knowledge has investigated how the infant joint attention and the parent's perception of the infant relate with language development across the acquisition of upright locomotion. It is essential to examine changes in such skills in order to chart the unfolding trajectory of these domains as function of locomotor experience. Parent report is a useful tool for providing researchers insight on variables warranting closer examination. The present longitudinal study incorporated the use of parent report of the above processes to explore how changes in infant social development across this transition relate with changes in infant language.

The aims of the investigation were two-fold. The first aim was to examine changes in the infant's social context across the transition from crawling to walking. Specifically, parents reported on infant initiating and following of joint attention behaviors, their perception of the infant as an intentional individual, and the infant's receptive and productive language. Use of parent report to measure these behaviors allowed for more frequent assessments across this developmental transition. It was hypothesized that infant initiation and following of joint attention behaviors would increase as a function of locomotor development, independent of age. It was also hypothesized that parents would perceive their infant as more responsible and intentional across the transition from crawling to walking. The second aim examined how changes in infant's social contexts across this motoric transition uniquely predicted language development over time as a function of walking experience. Parent–infant joint engagement, but not the parent's perception of the infant as an individual, was hypothesized to predict infant language controlling for infant age. Additionally, infant walking experience was expected to remain a unique predictor of receptive and productive language in this model.

## Methods

### Sample

Forty-three infants (24 female) were included in the present study, beginning when the infant was either 10 months (*n* = 17) or 10.5 months (*n* = 26) old and ending when the infant was 13.5 months of age. This sample was taken from a longitudinal study investigating infant language development and included language data previously reported in Study 1 by Walle and Campos (2014)^1^. This project was approved by the Committee for Protection of Human Subjects, University of California, Berkeley. Infants were predominantly from English-speaking families and heard English for a large proportion of the day. Extensive details regarding the demographics, backgrounds, and language environments of the sample are included in the report by Walle and Campos (2014). Forty infants were crawling at the start of the study (*M* age of crawling onset = 8.33 months, *SD* = 1.44) and three infants were walking at the start of the study (Age of walk onset = 9.63, 9.86, and 10.49 months, respectively).

### Procedure

Parents were emailed instructions for completing an online questionnaire administered using Qualtrics survey software. The email was sent to parents every 2 weeks, beginning when their child was 10- or 10.5-months-old and ending when their infant reached 13.5 months. The parent had 5 days to complete each online questionnaire, after which the link in the email was deactivated.

### Measures

The bi-weekly online questionnaire consisted of multiple surveys. The entire questionnaire was completed at each time point. The instructions at the start of the survey stated that the purpose of the study was to “investigate infant language and social development between 10 and 14 months of age.” No mentioning of the hypotheses relating to locomotor development was made.

Parents first completed a locomotor survey to indicate when their child had achieved specific locomotor milestones. Crawling onset was operationalized as the date when the infant could self-locomote a distance at least twice his or her body length. Walking onset was operationalized as the date when the infant first bipedally locomoted a distance of 10 feet without falling or needing support (see Adolph, 1997; Adolph et al., 2003). Previous research indicates high validity of parent reporting of infant motor milestones (e.g., Bodnarchuk and Eaton, 2004). No parents reversed their reporting of the onset of a locomotor transition.

Next, the parent completed the MacArthur-Bates Long Form Vocabulary Checklist: Level I (MCDI; Fenson et al., 1994). This survey contains a 396-item checklist in which parents marked words that the infant “understood” (receptive vocabulary) or “understood and says” (productive vocabulary). Parents were permitted to report their child's language development in any language, including signing. Items that the parent marked at previous time points were carried over into subsequent time points. The survey also includes a 12-item section on infant communicative gesturing. Validity and test–retest reliability for the MCDI is reported by Fenson et al. (1994).

Finally, parents completed a series of questions concerning their infants' social development. The questions asked parents to report on: infant pointing, infant bringing an object to the parent, infant point following, infant gaze following, and the parent's perception of the infant as an intentional individual responsible for his/her actions (see Appendix Section). For each question, the parent reported whether the behavior/perception of the infant was demonstrated significantly less, less, about the same, more, or significantly more during the most recent 2-week period in comparison to its frequency in the previous 2-week period. Parent reporting was scored on a scale of –2 (significantly <2 weeks ago) to 2 (significantly >2 weeks ago) to reflect the development of the particular item. Parent ratings at each interval were added cumulatively to reflect infant behavior and parent perception of the infant.

## Results

Initial analyses examined correlations between items relating to parent reporting of joint engagement behaviors and perception of their infant. The infant initiated behaviors of joint engagement (i.e., infant pointing, infant bringing object to parent) were highly correlated (*r* = 0.83, *p* < 0.001) and thus were combined into a composite variable named Infant Initiated Joint Engagement. Similarly, infant following of parent-initiated behaviors (i.e., infant following parent point, infant following parent gaze) were also correlated with one another (*r* = 0.57, *p* < 0.001) and were thus combined into a composite variable named Parent Initiated Joint Engagement. Finally, parent reporting of the infant as intentional and responsible for his/her actions were significantly correlated (*r* = 0.63, *p* < 0.001), and thus combined into a single variable named Infant as Individual. No effects of infant gender were observed, thus male and female infants were collapsed in all analyses.

### Analytic strategy

Mixed linear modeling using a first order autoregressive covariance structure was used to analyze change in variables across time. Infant Age and Walking Experience (i.e., number of weeks walking) were included in the models as fixed effects. Of the 296 reports analyzed, 27 contained missing values of parent reporting of infants' social development (9.12%). Visual inspection of the missing observations indicated no pattern of missingness, as the missing values were relatively evenly distributed across time points. Thus, instances of missing data were believed to be completely at random and resolved through imputation of the mean change score for the missing time point, a suitable solution given the circumstances of the present study (see Schafer and Graham, 2002).

Analysis of the skew and kurtosis of parent-reported variables indicated that the data was normally distributed at each time point.

The relation of Infant Age and Walking Experience with each of the three parent reported variables of infant social development (i.e., Infant Initiated Joint Engagement, Parent Initiated Joint Engagement, Infant as an Individual) was analyzed using separate mixed linear models for each social development variable (see Table 1). Next, a mixed linear model including Infant Age, Walking Experience, Infant Initiated Joint Engagement, Parent Initiated Joint Engagement, and Infant as an Individual examined the unique relation of these variables with infant (a) Receptive Vocabulary and (b) Productive Vocabulary (see Table 2).

**Table 1:** **Mixed linear models predicting infant social variables**.

**Variable**	**Infant initiated joint engagement**		**Parent initiated joint engagement**		**Infant as an individual**	
	***b***	**(*SE*)**	***b***	**(*SE*)**	***b***	**(*SE*)**
Age	0.49^**^	(0.08)	0.48^**^	(0.06)	0.57^**^	(0.06)
Walking Experience	−0.48*^†^*	(0.33)	−0.54^*^	(0.25)	−0.30	(0.26)
Walking Experience^2^	0.37^*^	(0.17)	0.28^*^	(0.13)	0.15	(0.13)
Walking Experience^3^	−0.05^*^	(0.02)	−0.03^*^	(0.02)	−0.01	(0.02)

*Values represent unstandardized fixed effect estimates and corresponding standard errors. Examination of non-linear trends as follows: Linear, Walking Experience; quadratic, Walking Experince^2^; cubic, Walking Experience^3^*.

† *p < 0.10*,

* *p < 0.05*,

** *p < 0.01*.

**Table 2:** **Full mixed linear model predicting infant MCDI scores**.

**Variable**	**Receptive MCDI**		**Productive MCDI**	
	***b***	**(*SE*)**	***b***	**(*SE*)**
Age	12.33^**^	(1.13)	3.49^**^	(0.49)
Walking Experience LN	16.53^**^	(3.57)		
Walking Experience			3.99^*^	(1.83)
Walking Experience^2^			−1.39	(0.93)
Walking Experience^3^			0.29^*^	(0.13)
Parent initiated joint engagement	3.88^**^	(1.35)	0.18	(0.57)
Infant as an individual	−3.02^*^	(1.25)	−0.59	(0.53)

*Values represent unstandardized fixed effect estimates and corresponding standard errors. Examination of non-linear trends were as follows: logarithmic, Walking Experience LN; linear, Walking Experience; quadratic, Walking Experince^2^; cubic, Walking Experience^3^*.

†*p < 0.10*,

* *p < 0.05*,

** *p < 0.01*.

### Data transformations

**Figure 1:**
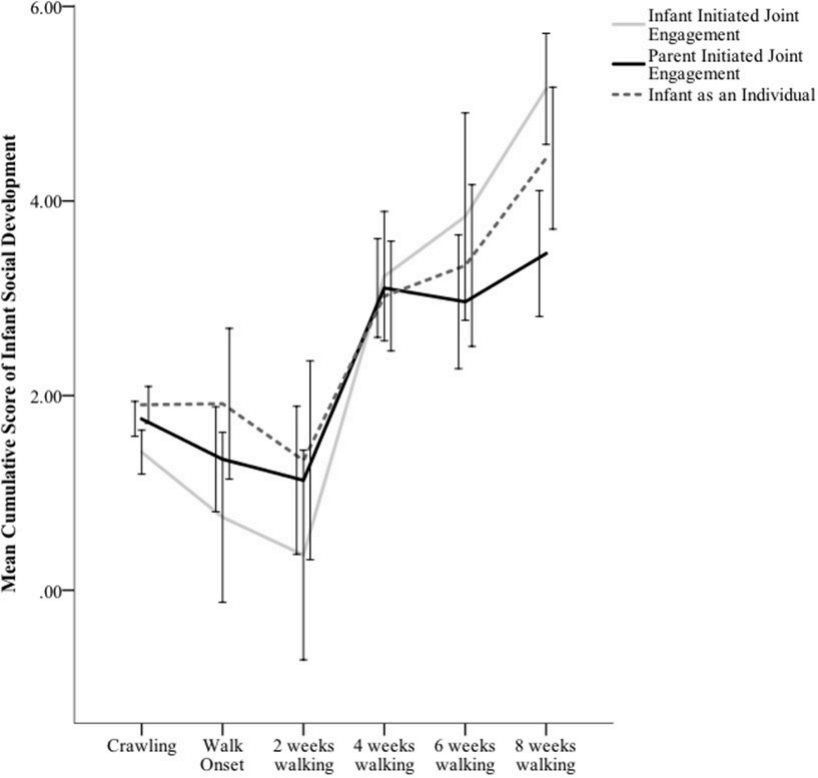
**Mean cumulative scores of parent reporting of Infant Initiation of Joint Engagement, Parent Initiation of Joint Engagement, and the parent's perception of the Infant as an Individual as a function of infant walking experience**. Error bars represent ± 1 *SE* of the mean.

As highlighted in the introduction, development is often non-linear, particularly when examined across a developmental transition. Visual inspection of changes in parent reporting of Infant Initiated Joint Engagement, Parent Initiated Joint Engagement, and Infant as an Individual as a function of Walking Experience suggested the presence of a non-linear, cubic trend (see Figure 1). Thus, Walking Experience was transformed using a cubic function (Walking Experience^3^) to test for the presence of this non-linear pattern of change (accordingly, a quadratic function, Walking Experience^2^, was also computed).

Additionally, in accordance with the previous reporting of the data by Walle and Campos (2014), the natural log of Walking Experience (Walking Experience LN) was used when predicting Receptive Vocabulary and the cubic function of Walking Experience (Walking Experience^3^) was used when predicting Productive Vocabulary. An extensive rationale for selecting these non-linear functions is provided on page 339 of the report by Walle and Campos (2014), specifically that the inclusion of the natural log and cubic functions of walking experience significantly improved the fit of models predicting receptive and productive language, respectively.

### Relations between variables of infant social development

An initial set of analyses examined the relations of Infant Initiated Joint Engagement, Parent Initiated Joint Engagement, and Infant as an Individual. Because each of these variables each assessed an aspect of infants' social development, these variables were expected to be related, yet representative of theoretically distinct constructs. The correlations of the variables were examined at each time point. The correlations of Infant Initiated Joint Engagement and Parent Initiated Joint Engagement were: *r*_*T*2_ = 0.32, *r*_*T*3_ = 0.64, *r*_*T*4_ = 0.65, *r*_*T*5_ = 0.71, *r*_*T*6_ = 0.79, *r*_*T*7_ = 0.80, *r*_*T*8_ = 0.84. The correlations of Infant Initiated Joint Engagement and Infant as an Individual were: *r*_*T*2_ = 0.32, *r*_*T*3_ = 0.64, *r*_*T*4_ = 0.65, *r*_*T*5_ = 0.71, *r*_*T*6_ = 0.79, *r*_*T*7_ = 0.80, *r*_*T*8_ = 0.84. The correlations of Parent Initiated Joint Engagement and Infant as an Individual were: *r*_*T*2_ = 0.51, *r*_*T*3_ = 0.66, *r*_*T*4_ = 0.60, *r*_*T*5_ = 0.55, *r*_*T*6_ = 0.59, *r*_*T*7_ = 0.62, *r*_*T*8_ = 0.62. All of the above correlations were significant (*p* < 0.05). Although the variables were correlated with one another, prior theoretical considerations viewed each as a separate construct. Thus, each variable was analyzed separately to examine their relation with infant walking experience. However, analyses examining the unique associations of the variables with infant language included only Parent Initiated Joint Engagement and Infant as an Individual, as these two variables were the least associated (see below).

### Infant initiated joint engagement and infant walking

Change in Infant Initiated Joint Engagement was examined as a function of Infant Age and Walking Experience. An initial model including only Age and Walking Experience was first tested. A significant effect of Age was present, *t*_(277)_ = 6.11, *p* < 0.001, β = 0.29, but Walking Experience was only trending, *t*_(279)_ = 1.84, *p* = 0.067, β = 0.10.

Next, a model was tested including the cubic function of Walking Experience. As shown in Table 1, significant effects of infant Age, *t*_(275)_ = 6.23, *p* < 0.001, β = 0.29, the quadratic effect Walking Experience^2^, *t*_(229)_ = 2.27, *p* = 0.02, β = 0.64, and the cubic effect Walking Experience^3^, *t*_(229)_ = 2.01, *p* = 0.045, β = 0.35, were present. The linear effect of Walking Experience did not reach significance, *t*_(237)_ = 1.47, *p* = 0.14, β = 0.20. Although this model seemed to better reflect the pattern of development indicated by the graphing of the data, this model did not demonstrate a significantly better fit than the linear model, χ^2^(1) = 0.55, *p* = 0.46.

Graphing the changes in Infant Initiated Joint Engagement suggested differences between 2- and 4-weeks and 4- and 6-weeks of walking experience (see Figure 1). Pairwise comparisons controlling for Infant Age revealed significant differences in Infant Initiated Joint Engagement between 2-weeks (*M* = 1.55, *SE* = 0.55) and 4-weeks (*M* = 2.29, *SE* = 0.59), *t*_(245)_ = 2.91, *p* = 0.004, 95% CI [−1.24, −0.24], and 4- and 6-weeks (*M* = 2.90, *SE* = 0.65) after the onset of walking, *t*_(244)_ = 2.18, *p* = 0.03, 95% CI [−1.17, −0.06].

### Parent initiated joint engagement and infant walking

Change in infant following of Parent Initiated Joint Engagement was examined as a function of Infant Age and Walking Experience. An initial model including only Age and Walking Experience was first tested. A significant effect of Age was present, *t*_(278)_ = 8.01, *p* < 0.001, β = 0.39, but Walking Experience was not significant, *t*_(279)_ = 0.04, *p* = 0.97, β = 0.00.

Next, a model was tested including the cubic function of Walking Experience. As shown in Table 1, significant effects of infant Age, *t*_(276)_ = 8.09, *p* < 0.001, β = 0.39, the linear effect of Walking Experience, *t*_(237)_ = 2.18, *p* = 0.03, β = 0.30, the quadratic effect Walking Experience^2^, *t*_(228)_ = 2.24, *p* = 0.03, β = 0.65, and the cubic effect Walking Experience^3^, *t*_(228)_ = 2.00, *p* = 0.046, β = 0.36, were present. Again, although the cubic model appeared to reflect the visual patterning of the data, this model did not demonstrate a significantly better fit than the linear model, χ^2^(1) = 0.48, *p* = 0.49.

Graphing the changes in Parent Initiated Joint Engagement suggested differences between 2- and 4-weeks (see Figure 1). Pairwise comparisons controlling for Infant Age confirmed the visual pattern, revealing significant differences between 2-weeks (*M* = 1.52, *SE* = 0.40) and 4-weeks (*M* = 1.98, *SE* = 0.43) after the onset of walking, *t*_(245)_ = 2.34, *p* = 0.02, 95% CI [−0.84, −0.09].

### Infant as an individual and infant walking

Change in the Infant as an Individual was examined as a function of Infant Age and Walking Experience. An initial model including only Age and Walking Experience was first tested. A significant effect of Age was present, *t*_(276)_ = 8.94, *p* < 0.001, β = 0.40, but Walking Experience was not significant, *t*_(278)_ = 0.84, *p* = 0.40, β = 0.04.

Next, a model was tested including the cubic function of Walking Experience. As shown in Table 1, a significant effect of infant Age, *t*_(274)_ = 9.00, *p* < 0.001, β = 0.40, was present. However, neither the linear effect of Walking Experience, *t*_(236)_ = 1.16, *p* = 0.25, β = 0.15, the quadratic effect Walking Experience^2^, *t*_(229)_ = 1.14, *p* = 0.26, β = 0.30, nor the cubic effect Walking Experience^3^, *t*_(229)_ = 0.73, *p* = 0.47, β = 0.12, were significant. Additionally, this model demonstrated worse fit than the linear model, χ^2^(1) = −1.72, *p* < 0.001.

As with the previous two parent-reported variables, graphing of changes of the Infant as an Individual suggested possible differences as a function of infant Walking Experience (see Figure 1). Thus, for exploratory purposes, pairwise comparisons controlling for Infant Age examined this interval. Analyses revealed significant differences between 2-weeks (*M* = 1.80, *SE* = 0.46) and 4-weeks (*M* = 2.24, *SE* = 0.49) after the onset of walking, *t*_(244)_ = 2.18, *p* = 0.03, 95% CI [−0.84, −0.04].

### Relations with language

All of the above variables were included in a single mixed linear model to examine their unique relations with infant receptive and productive vocabulary (see Table 2). As with the prior models, infant Age and Walking Experience were also included to determine whether the relations reported by Walle and Campos (2014) would remain significant with inclusion of the variables from the present study. Additionally, although Infant Initiated Joint Engagement and Parent Initiated Joint Engagement were theorized to be unique constructs, they were also highly correlated, *r* = 0.79, *p* < 0.001. Thus, to avoid issues of collinearity, only Parent Initiated Joint Engagement was included in models predicting infant language. The selection for including Parent Initiated Joint Engagement was based on this variable having a smaller correlation with Infant as an Individual than did Infant Initiated Joint Engagement.

#### Receptive vocabulary

The effects of Parent Initiated Joint Engagement, *t*_(249)_ = 2.87, *p* = 0.004, β = 0.16, and parent reporting of the Infant as an Individual, *t*_(251)_ = 2.41, *p* = 0.02, β = 0.14, each predicted infant receptive vocabulary size. Additionally, infant Age, *t*_(243)_ = 10.96, *p* < 0.001, β = 0.41, and Walking Experience LN, *t*_(236)_ = 4.63, *p* < 0.001, β = 0.16, remained significant predictors of receptive vocabulary.

#### Productive vocabulary

For infant productive vocabulary, only significant effects for infant Age, *t*_(250)_ = 7.10, *p* < 0.001, β = 0.40, Walking Experience, *t*_(209)_ = 2.18, *p* = 0.03, β = 0.31, and the cubic function Walking Experience^3^, *t*_(200)_ = 2.27, *p* = 0.02, β = 0.43, were present. However, Parent Initiated Joint Engagement, *t*_(247)_ = 0.32, *p* = 0.75, β = 0.03, and the Infant as an Individual, *t*_(243)_ = 1.13, *p* = 0.26, β = 0.10, were not significant, nor was the quadratic function Walking Experience^2^, *t*_(200)_ = 1.50, *p* = 0.14, β = 0.46.

## Discussion

Parent reporting indicated that infant elicitation and following of parent attention increases following the onset of walking, independent of the infant's age. These infant behaviors have not previously been linked with the onset of walking. Interestingly, parents' perception of their infant did not increase as a function of walking experience; only as a function of age. Importantly, the relation of infant walking and language remained significant even after controlling for infant joint attention engagement and the parents' perception of the infant. This suggests that (1) parental bias is unlikely to account for the reported differences in walking and crawling infants' language development, and (2) the study of additional developmental domains, particularly immediately after the transition from crawling to walking, is needed to further explore the association of walking and language. Each of these findings is elaborated upon below and suggestions for further research are described.

### Infant and parent behaviors across the transition from crawling to walking

#### Infant and parent initiated joint engagement

Examination of the longitudinal data revealed that significant increases in infant initiation and following of joint engagement between 2- to 4-weeks and 4- to 6-weeks following the onset of walking. Importantly, these differences were present independent of infant age. Parent reported changes in infants' initiation of joint engagement across the transition from crawling to walking mirrors prior observational findings (e.g., Clearfield et al., 2008; Clearfield, 2011; Karasik et al., 2014). The present results extend previous research by demonstrating the relation of these behaviors with infant concurrent vocabulary size across this transition. Further research is needed to follow up on this finding in two additional contexts. Furthermore, these findings provide support for the possibility that changes in infant following of joint attention cues reported at around 12 months of age may be related to infant walking onset. For example, longitudinal research by Morissette et al. (1995) reported nearly a 70% increase in infants' following of adult gaze to locate a referent between 12 and 15 months of age, likely when most infants had shifted from crawling to experienced walkers.

#### Parent perception of infant as an individual

Contrary to our hypotheses, the onset of walking did not significantly impact the parent's perception of the infant. Even so, it remains important to examine possible qualitative differences in parent speech to walking infants. Both Walle and Campos (2014) and Walle and Warlaumont (2015) found that parent language input predicted walking, but not crawling, infants' language development, despite walking and crawling infants receiving similar amounts of language input. Additionally, Karasik et al. (2014) found that parents were more likely to respond to infant mobile bids (i.e., carrying an object to the parent) with action directives related to the infants' object of interest. Although mobile bids were more frequent by walking infants, parents demonstrated a similar style of responding when crawling infants engaged in this behavior, suggesting that the parents' perception of the infant in of itself may not impact language input. However, this does not rule out that walking and crawling infants may qualitatively differ in their processing of such input.

### Predicting infant language development

Inclusion of the above variables with infant age and walking experience allowed for the examination of the unique relation of the predictors with infant receptive and productive language development.

#### Receptive vocabulary

Infant following of parent-initiated episodes of joint engagement was positively related with receptive vocabulary size, independent of infant age and locomotor ability. Interestingly, the relation of infant walking with infant receptive language remained significant, though the coefficient did drop from *b* = 35.65, 95% CI [19.48, 51.82] (as reported by Walle and Campos, 2014, in which the same data was analyzed without the parent-reported social variables) to *b* = 16.53. This suggests that while social engagement behaviors are important for the development of infant language (e.g., Tomasello and Todd, 1983; Iverson and Goldin-Meadow, 2005), their role does not fully account for the relation of infant walking and language. Additionally, the parent's perception of the infant as an individual also significantly predicted receptive language. However, the negative direction of this relation suggests that parents may, in fact, deflate their reporting of infant's receptive language across this transition. Furthermore, parents' perception of the infant as an intentional and responsible individual did not account for the relation of infant walking and language. Though by no means definitive, this finding provides evidence against the possibility that parental bias in reporting infants' language development for crawling and walking infants may account for previous findings reported by He et al. (2015) and Walle and Campos (2014).

#### Productive vocabulary

Contrary to the hypotheses, joint engagement behaviors were not related to infant productive vocabulary size. Additionally, parent reporting of infant productive language was not influenced by the parent's perception of the infant as an individual. However, as with receptive language, the relation of infant walking experience was a significant predictor of infant productive vocabulary size. These findings may indicate that productive language development across the transition from crawling to walking is impacted by different mechanisms than those included in the present study. For example, whereas receptive language may be aided by increased adult labeling of objects in the environment during episodes of joint engagement, increases in productive language may be facilitated by other means. For example, physiological changes resulting from an upright posture, such as changes in respiration, positioning of the diaphragm, or length of the vocal tract (Openshaw et al., 1984; Thelen, 1991; see Boliek et al., 1996; Vorperian et al., 2005), may facilitate ease of verbalization and articulation. Additionally, increased motoric coordination more generally, fundamental for both walking and speech production (see Iverson, 2010), may also account for the relation.

### Developmental patterning of change

The findings from the present investigation also revealed parent reporting of infant initiation and following of joint attention cues significantly increased between 2- and 4-weeks after the infant began to walk. This suggests that changes related to the acquisition of walking may necessitate multiple weeks before manifesting. This finding is similar to the longitudinal findings by Walle and Campos (2014) for infant productive vocabulary. Additionally, similar delays in functional change has been observed across other developmental transitions (e.g., Campos et al., 1992; Eilers et al., 1993; Bertenthal et al., 1994). Future research comparing crawling and walking infants may wish to allow a sufficient amount of walking experience when predicting corresponding developmental change in various domains. Additionally, it is necessary to more closely examine what occurs during the first 4 weeks following the acquisition of infant walking. It has been hypothesized that developmental transitions correspond with a temporary reorganization of various skills as the system adjusts to the new skill and related experiences (see Thelen and Smith, 1994) and empirical research lends some support to this notion (e.g., Clearfield, 2004; Berger, 2010; Parladé and Iverson, 2011). It is possible that infant engagement with the social environment immediately following the onset of walking is hampered due to a need for increased allocation of attention to postural stability. Thus, the benefits afforded by upright locomotion may only be gleaned after sufficient expertise for the new locomotor skill is achieved.

## Limitations for consideration

Although the present study found associations between parent and infant social behaviors and the infant's language development, there are two notable limitations that warrant acknowledgement.

First and foremost, all data collected relied on parent reporting of infant behavior and development and laboratory and observational assessment of the variables reported in the present study is needed. Laboratory assessments of infant elicitation of adult behavior (e.g., Harding and Golinkoff, 1979; Lempers, 1979; Conrad, 1994) would more precisely examine differences in crawling and walking infants' initiation of joint engagement. Additionally, paradigms similar to those by Butterworth and others (see Butterworth and Cochran, 1980; Butterworth and Jarrett, 1991) in which the adult attempts to direct the infant's attention to novel objects is needed to more carefully observe the parent reported differences found in the present study. In particular, it would be of interest to test infant following of a variety of different communicative cues (Presmanes et al., 2007), particularly adult gaze (Morissette et al., 1995), and infant locating of referents outside of their immediate visual field (Deák et al., 2000). Naturalistic observations in which the parent and child are observed regularly in the home for extensive periods of time would help to corroborate changes in infant initiated joint engagement behaviors, how parents respond to such behaviors, and the relation of the behaviors and interactions with concurrent and subsequent infant language development (Iverson and Goldin-Meadow, 2005; Clearfield et al., 2008; Karasik et al., 2014). Furthermore, research examining the quality of parent language and the reciprocal patterning within the language environment (e.g., parent scaffolding of infant babbling, turn-taking, engagement with joint objects) is vital for furthering understanding the infants' language environments (see Warlaumont et al., 2014).

Second, although the longitudinal design of the study captured developmental change across the transition from crawling to walking, the data collected assessed perceived changes in infant joint engagement and independence, not actual values for these constructs. This more descriptive approach prevented the present study from determining the objective frequency or level of sophistication of infant behaviors. Furthermore, parents may have differed in their operationalization of certain behaviors, such as what they considered indicative of infant following of social cues or independence. What is clearly needed is a multi-method longitudinal investigation incorporating laboratory assessments, direct observation, and parent reporting to examine the developmental trajectories of these variables across the transition from crawling to walking. Such an investigation could feature (1) bi-weekly lab testing of infant joint attention, imitation, representation, receptive and productive language, and lab observation of parent-child interactions, (2) bi-weekly assessment of the home language environment (e.g., Walle and Warlaumont, 2015), and (3) continuous parent reporting of infant development using mobile technology (e.g., Ellis-Davies et al., 2012). Though expensive with regard to time and resources, an investigation of this sort is precisely what is required at this juncture to more precisely examine the relation of infant walking with other psychological skills.

In closing, it is important to highlight the typical fashion that research in developmental psychology often proceeds. First, one identifies a developmental change through observation. Second, one investigates relations of the developmental change and other relevant variables. Third, one engages in more precise testing of the identified variables to establish a causal relation. The present investigation provides important information relevant to the second step that is intended to inform the third. Although a causal association between infant walking and language has not be demonstrated by existing research, the antecedent-consequent nature of the findings supports a view favoring epigenesis over maturational coincidence. Continued research is needed to (1) replicate the relation of walking and language and (2) identify possible mediators or moderators of this relation. Such work would further our understanding of the complex relation of walking with other psychological phenomena.

## Author contributions

The author confirms being the sole contributor of this work and approved it for publication.

### Conflict of interest statement

The author declares that the research was conducted in the absence of any commercial or financial relationships that could be construed as a potential conflict of interest.

## Footnotes

^1^Data from 1 infant of the sample from Study 1 of Walle and Campos (2014) was excluded from the present study because the parent did not complete the questionnaire of changes in infant behavior.

## Appendix

### Parent questionnaire items

In the past 2 weeks, how frequently have the following happened?

**Table:**

My child pointed at an object or event that was of interest to him/her.^a^
My child brought me an object that was of interest to him/her.^a^
My child followed my pointing to an object or event of interest.^b^
My child followed my looking to an object or event of interest (without my pointing to it).^b^
I had the clear sense that my child was intentionally acting on his/her environment.^c^
I felt that my child should be held responsible for his/her actions.^c^

Each items rated on a 5-point scale, anchored by “Much Less than 2 weeks ago” and “Much more than 2 weeks ago.” Superscripts denote items combined in subsequent analyses (

a Child initiated joint engagement;

b Parent initiated joint engagement;

c Infant as an Individual).
